# Supplementary material for: Eupatorin and Salviandulin-A, with Antimicrobial and Anti-Inflammatory Effects from Salvia lavanduloides Kunth Leaves
Source: Plants (Basel). 2022 Jun 30;11(13):1739. doi: 10.3390/plants11131739 (PMC9269164; doi:10.3390/plants11131739)
Supplement: Supplementary file 1 [file plants-11-01739-s001.zip › plants-1774599-supplementary.pdf]

plants

MDPI

Supplementary Data

Eupatorin and salviandulin-A, with antimicrobial and anti-inflammatory effect of *Salvia lavanduloides* Kunth leaves

Manasés González-Cortazar [1], David Osvaldo Salinas-Sánchez [2,3,*], Maribel Herrera-Ruiz [1], Dionisio Celerino Román-Ramos [3], Alejandro Zamilpa [1], Enrique Jiménez-Ferrer [1], Ever A. Ble-González [4], Patricia Álvarez-Fitz [5], Ricardo Castrejón-Salgado [6] and Ma. Dolores Pérez-García [1,*]

1 Centro de Investigación Biomédica del Sur, Instituto Mexicano del Seguro Social, Argentina No. 1, Col. Centro, Xochitepec 62790, Morelos, Mexico; gmanases2000@gmail.com (M.G.-C.); edanae10@yahoo.com.mx (M.H.-R.); azamilpa_2000@yahoo.com.mx (A.Z.); enriqueferrer_mx@yahoo.com (E.J.-F.)
2 Centro de Investigación en Biodiversidad y Conservación (CIByC), Universidad Autónoma del Estado de Morelos (UAEM), Av. Universidad 1001, Col. Chamilpa, Cuernavaca 62209, Morelos, Mexico
3 Escuela de Estudios Superiores del Jicarero (EESJ), Universidad Autónoma del Estado de Morelos, Carretera Galeana-Tequesquitengo s/n Col. el Jicarero, Jojutla 62909, Morelos, Mexico; roman.ramos.dionisio@gmail.com
4 División Académica de Ciencias Básicas, Universidad Juárez Autónoma de Tabasco, Carretera Cunduacán-Jalpa Km. 0.5, Cunduacán 86690, Tabasco, Mexico; ble_49@hotmail.com
5 Laboratorio de Toxicologia, Cátedra CONACyT-Universidad Autónoma de Guerrero, Av. Lázaro Cárdenas s/n. Col. La Haciendita, Chilpancingo 39070, Guerrero, Mexico; paty_fitz@hotmail.com
6 Instituto Mexicano del Seguro Social, Unidad de Medicina Familiar Número 3, Avenida Insurgentes Esquina con Emiliano Zapata s/n., Centro, Jiutepec 62550, Morelos, Mexico; carisal13@hotmail.com
* Correspondence: davidos@uaem.mx (D.O.S.-S.); lola_as@yahoo.com.mx (M.D.P.-G.); Tel.: +52-(777)-3297019 (D.O.S.-S.); +52-(777)-3612155 (M.D.P.-G.)

Content

Figure S1. ^{1}H NMR ($CDCl_3$, 600 MHz) of compound (**1**)

Figure S2. ^{13}C NMR ($CDCl_3$, 150 MHz) of compound (**1**)

Figure S3. ^{3}C (DEPT) NMR ($CDCl_3$, 150 MHz) of compound (**1**)

Figure S4. ^{1}H-^{1}H COSY NMR ($CDCl_3$, 600 MHz) of compound (**1**)

Figure S5. ^{1}H-^{13}C (HSQC) NMR ($CDCl_3$, 600 MHz) of compound (**1**)

Figure S6. ^{1}H-^{13}C (HMBC) NMR ($CDCl_3$, 600 MHz) of compound (**1**)

Figure S7. ^{1}H NMR ($CDCl_3$, 600 MHz) of salviandulin (**2**)

Figure S8. ^{13}C NMR ($CDCl_3$, 150 MHz) of salviandulin (**2**)

Figure S9. ^{3}C (DEPT) NMR ($CDCl_3$, 150 MHz) of salviandulin (**2**)

Figure S10. ^{1}H-^{1}H COSY NMR ($CDCl_3$, 600 MHz) of salviandulin (**2**)

Figure S11. ^{1}H-^{13}C (HSQC) NMR ($CDCl_3$, 600 MHz) of salviandulin (**2**)

Figure S12. ^{1}H-^{13}C (HMBC) NMR ($CDCl_3$, 600 MHz) of salviandulin (**2**)

Figure S13. ^{1}H NMR (CD_3COCD_3, 600 MHz) of eupatorin (**3**)

Figure S14. ^{13}C NMR (CD_3COCD_3, 150 MHz) of eupatorin (**3**)

Figure S15. ^{3}C (DEPT) NMR (CD_3COCD_3, 150 MHz) of eupatorin (**3**)

Figure S16. ^{1}H-^{1}H COSY NMR (CD_3COCD_3, 600 MHz) of eupatorin (**3**)

Figure S17. ^{1}H-^{13}C (HSQC) NMR (CD_3COCD_3, 600 MHz) of eupatorin (**3**)

Figure S18. ^{1}H-^{13}C (HMBC) NMR (CD_3COCD_3, 600 MHz) of eupatorin (**3**)

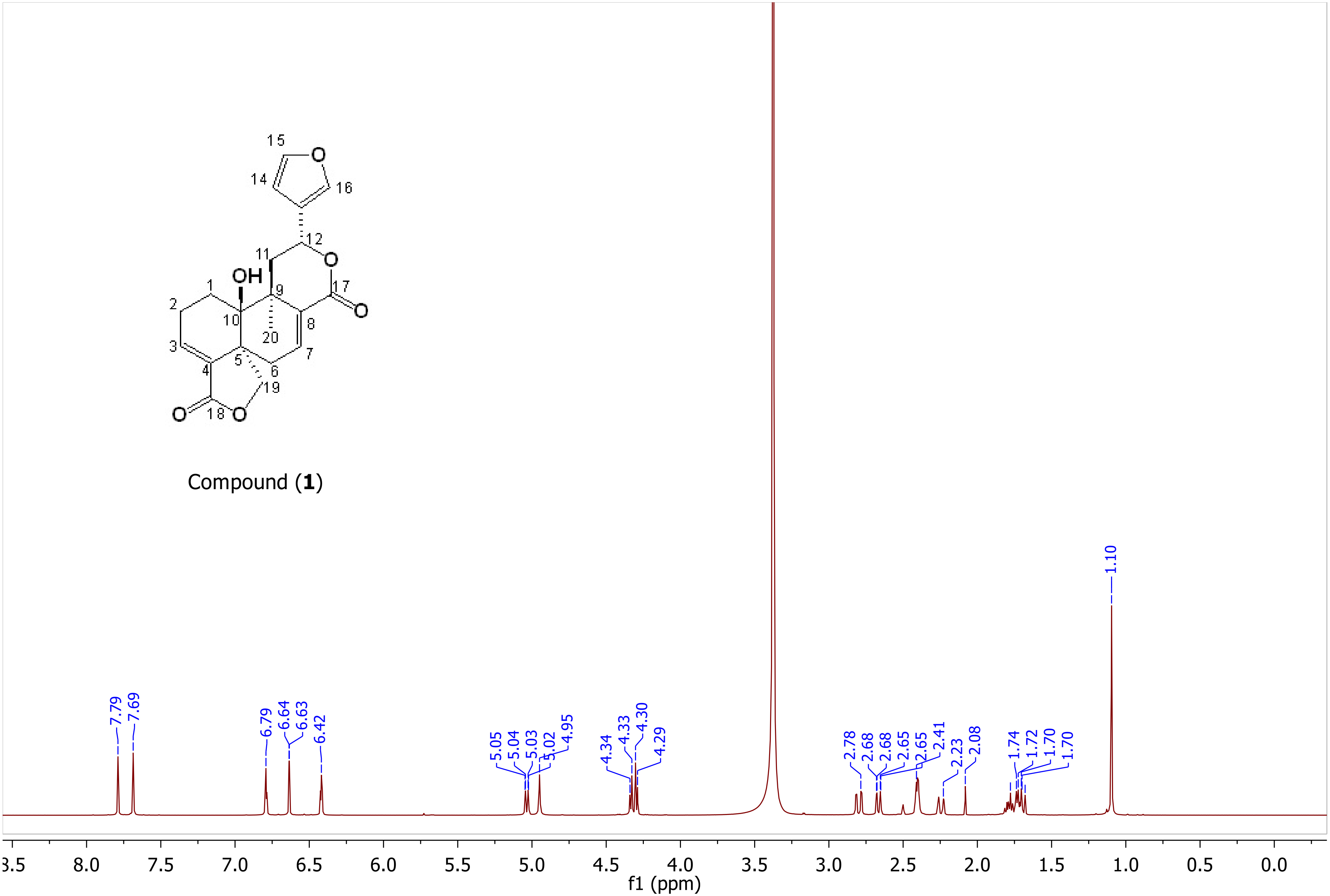

Figure S1. ^{1}H NMR ($CDCl_3$, 600 MHz) of compound (**1**)

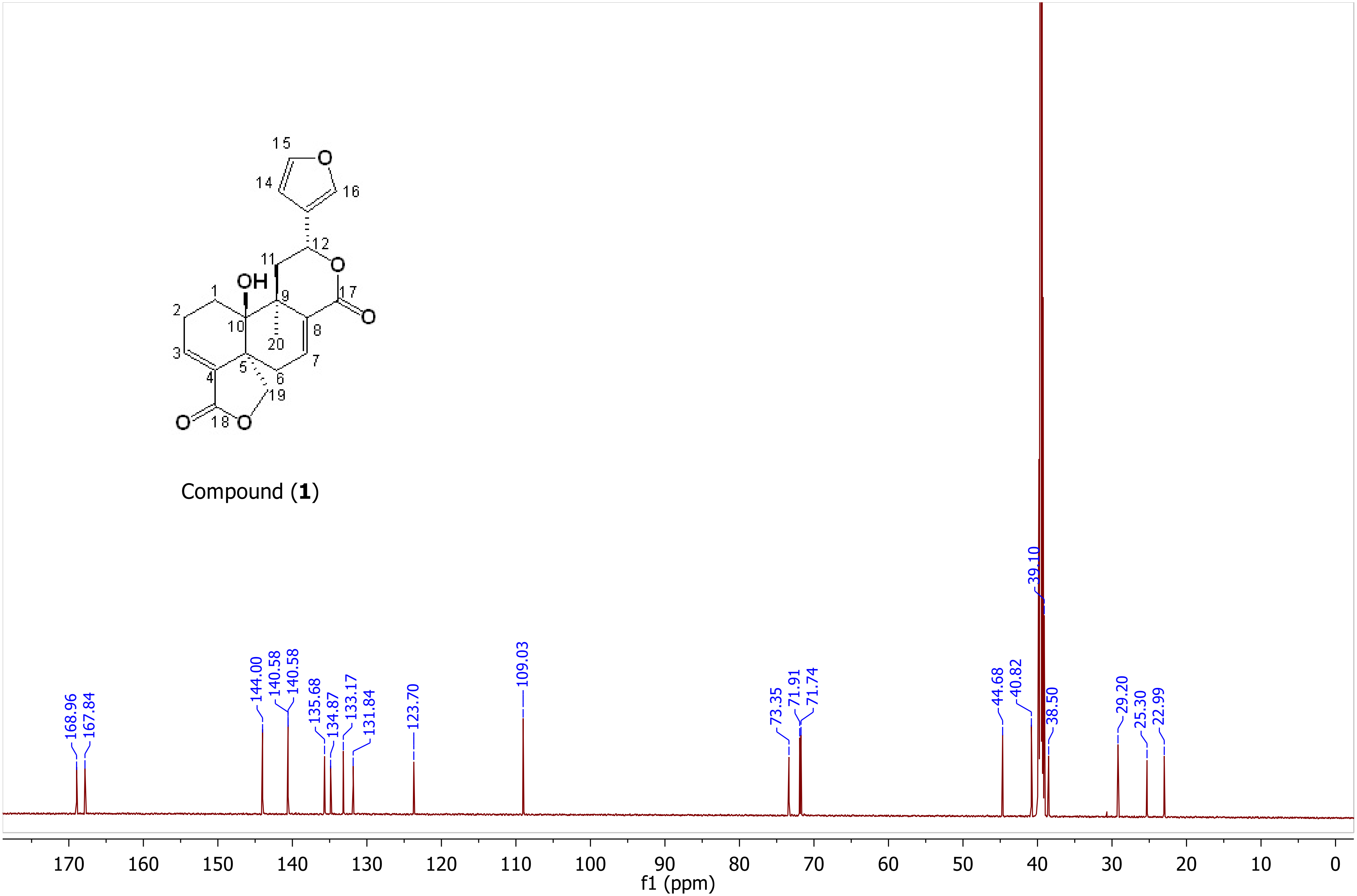

Figure S2. ^{13}C NMR ($CDCl_3$, 150 MHz) of compound (**1**)

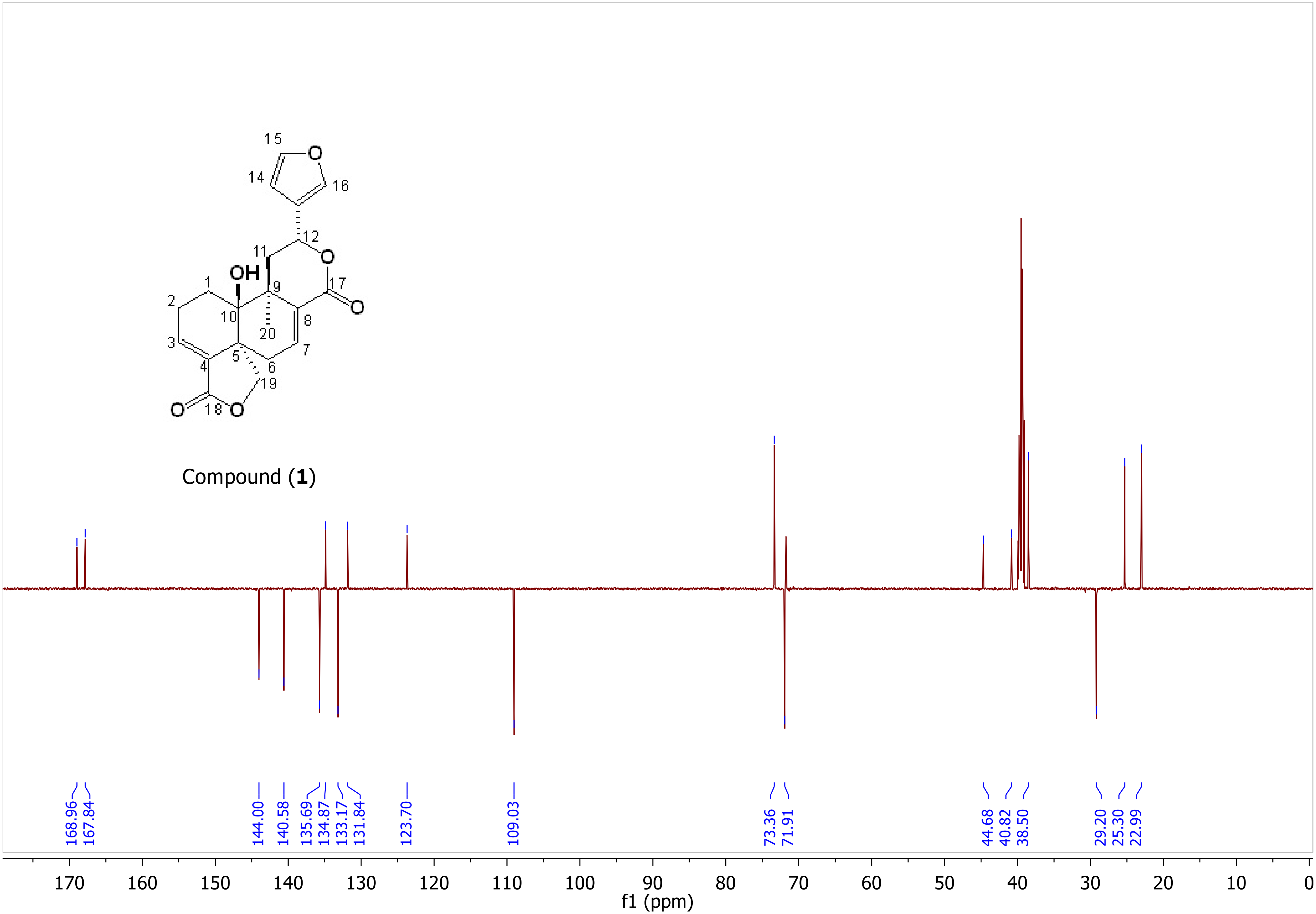

Figure S3. ^{13}C (DEPT) NMR ($CDCl_3$, 150 MHz) of compound (**1**)

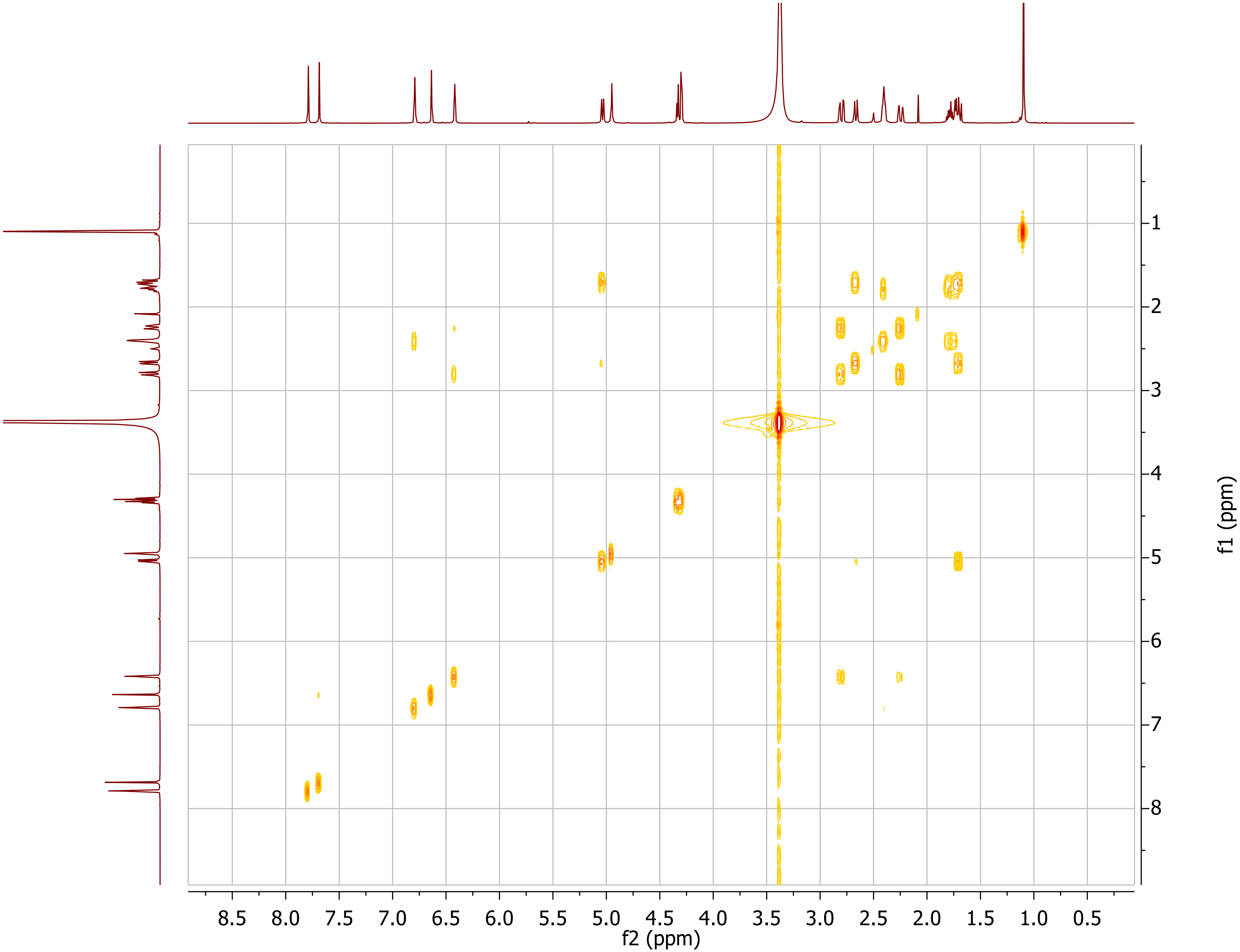

Figure S4. ^{1}H-^{1}H COSY NMR ($CDCl_3$, 600 MHz) of compound (**1**)

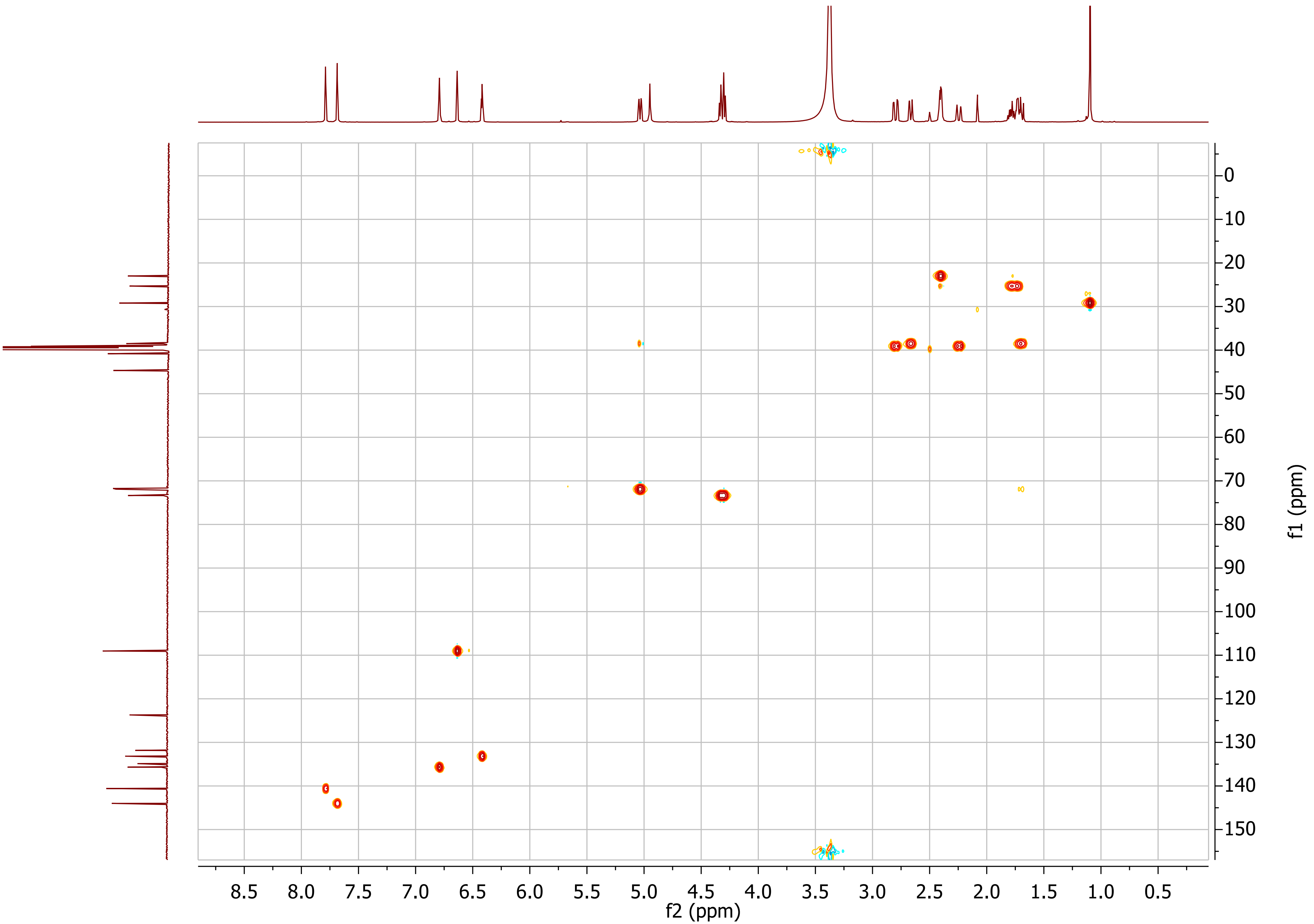

Figure S5. ^{1}H-^{13}C (HSQC) NMR ($CDCl_3$, 600 MHz) of compound (**1**)

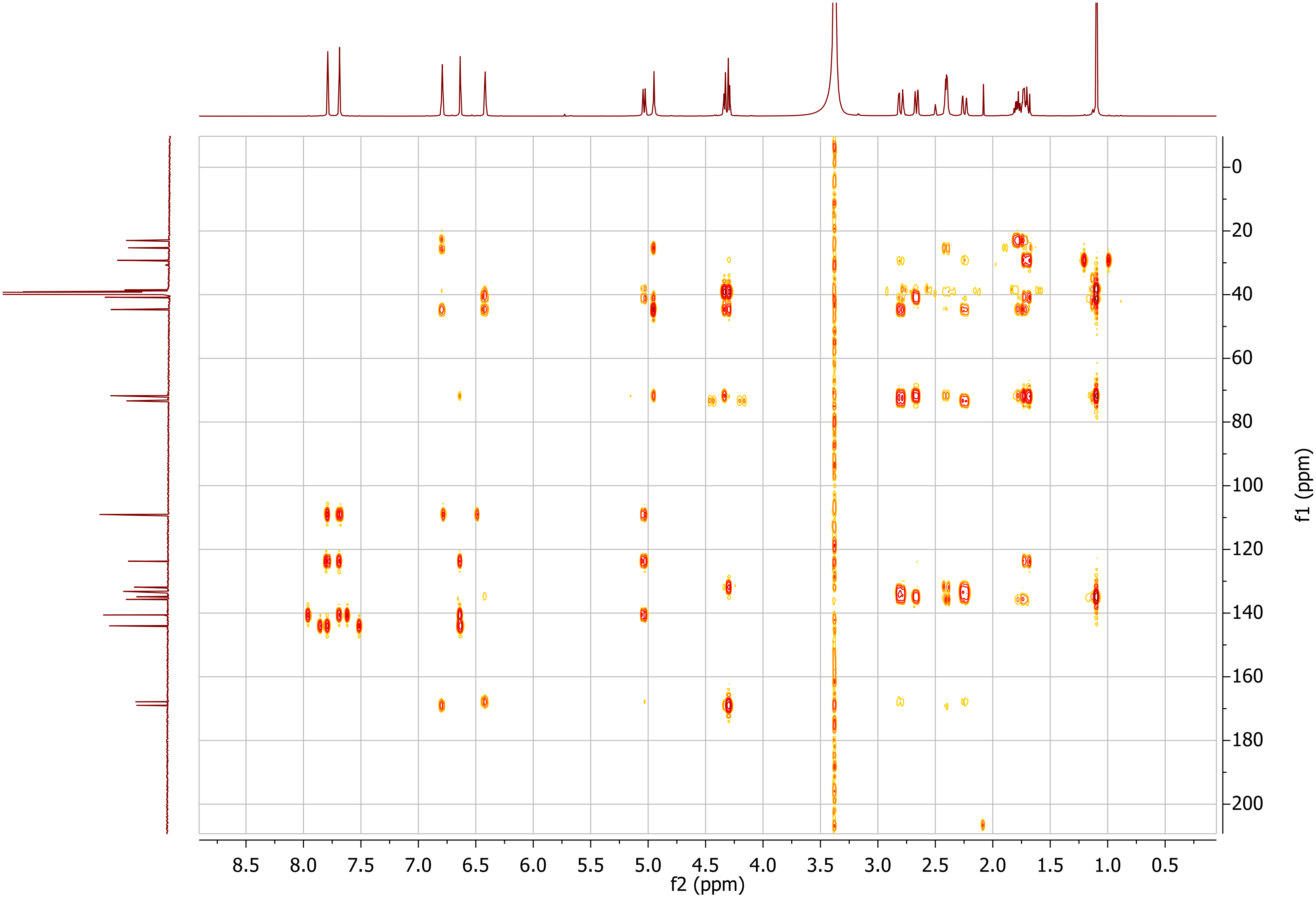

Figure S6. ^{1}H-^{13}C (HMBC) NMR ($CDCl_3$, 600 Mhz) of compound (**1**)

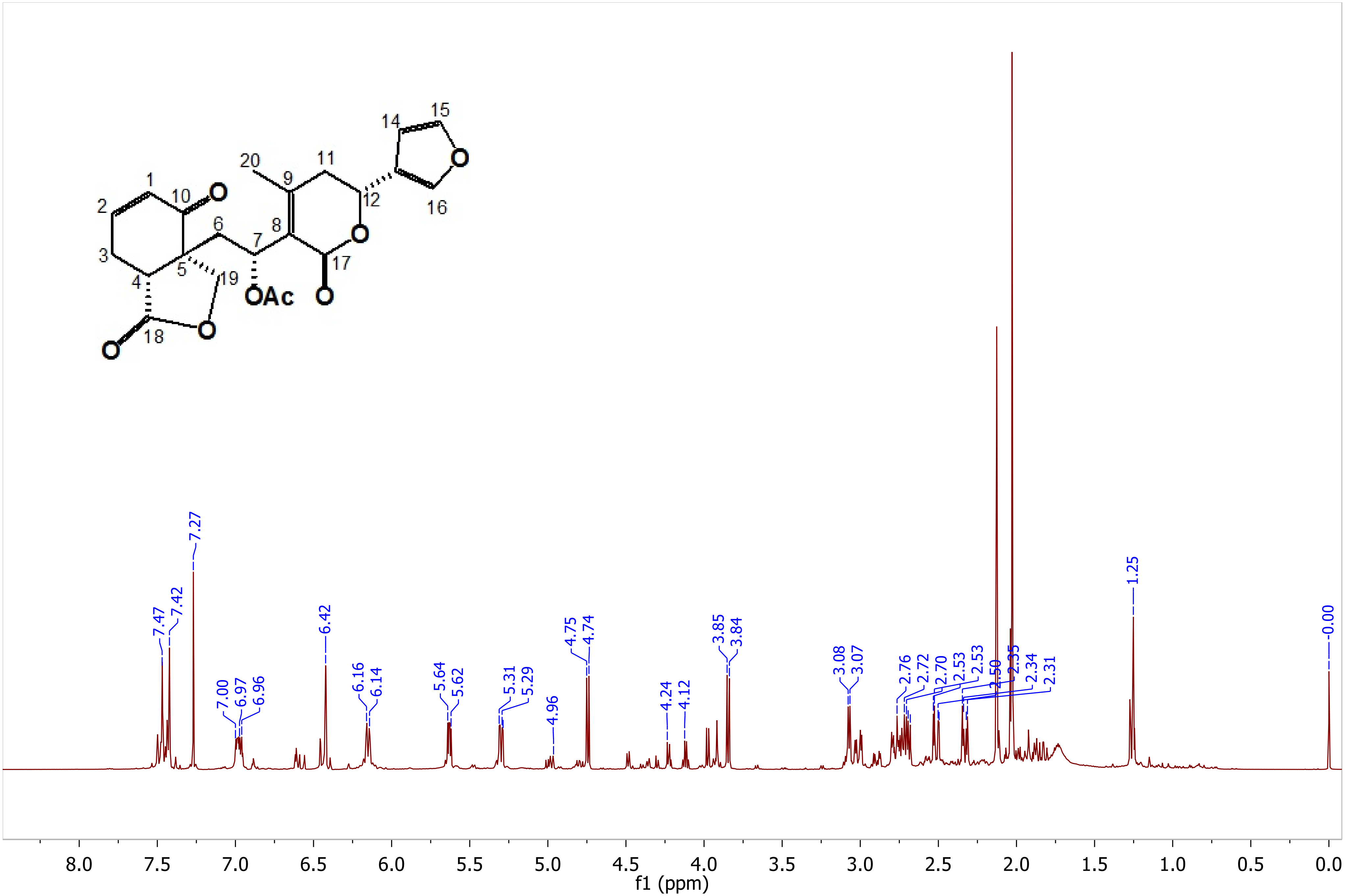

Figure S7. ^{1}H NMR ($CDCl_3$, 600 MHz) of salviandulin A (**2**)

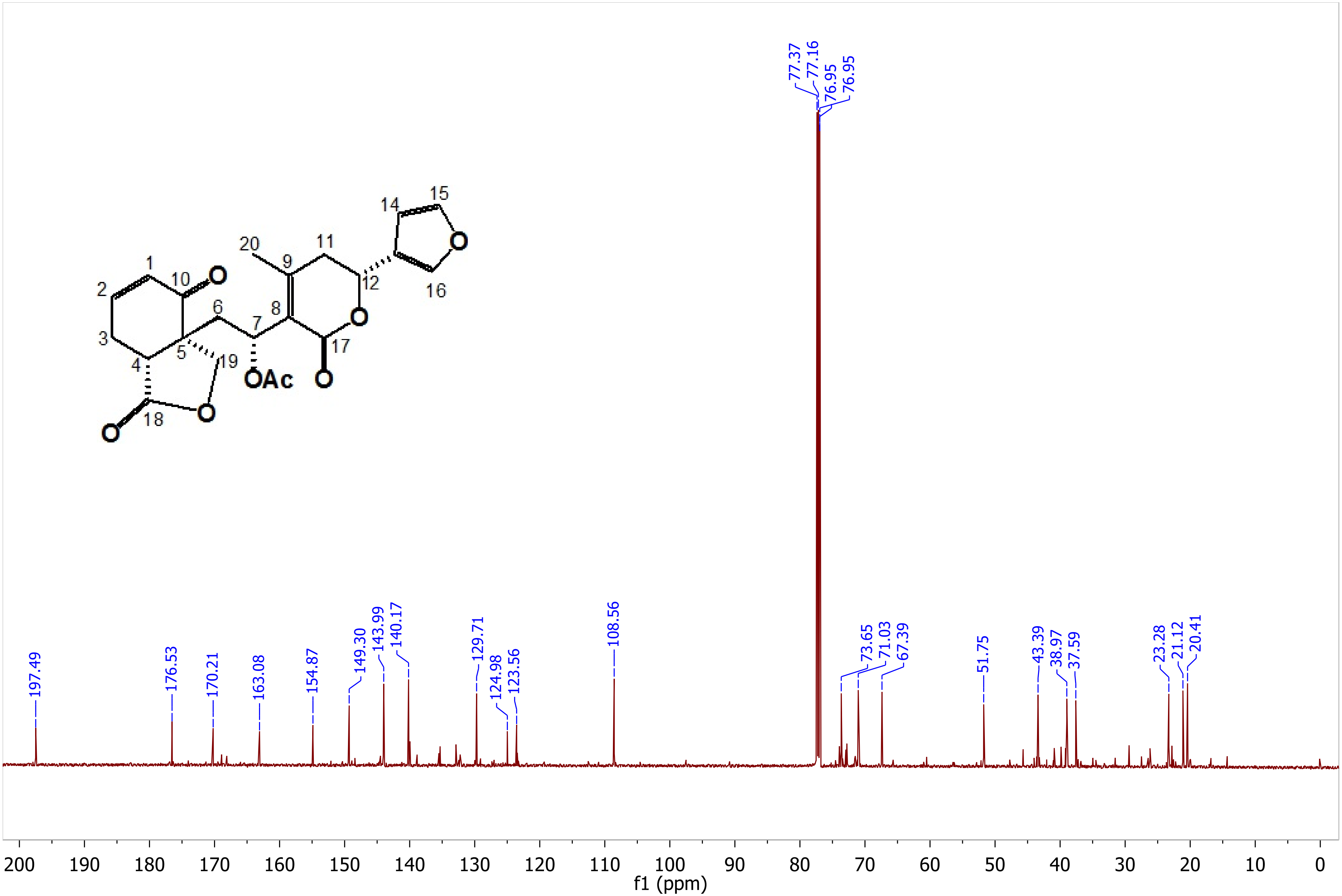

Figure S8. ^{13}C NMR ($CDCl_3$, 150 MHz) of salviandulin A (**2**)

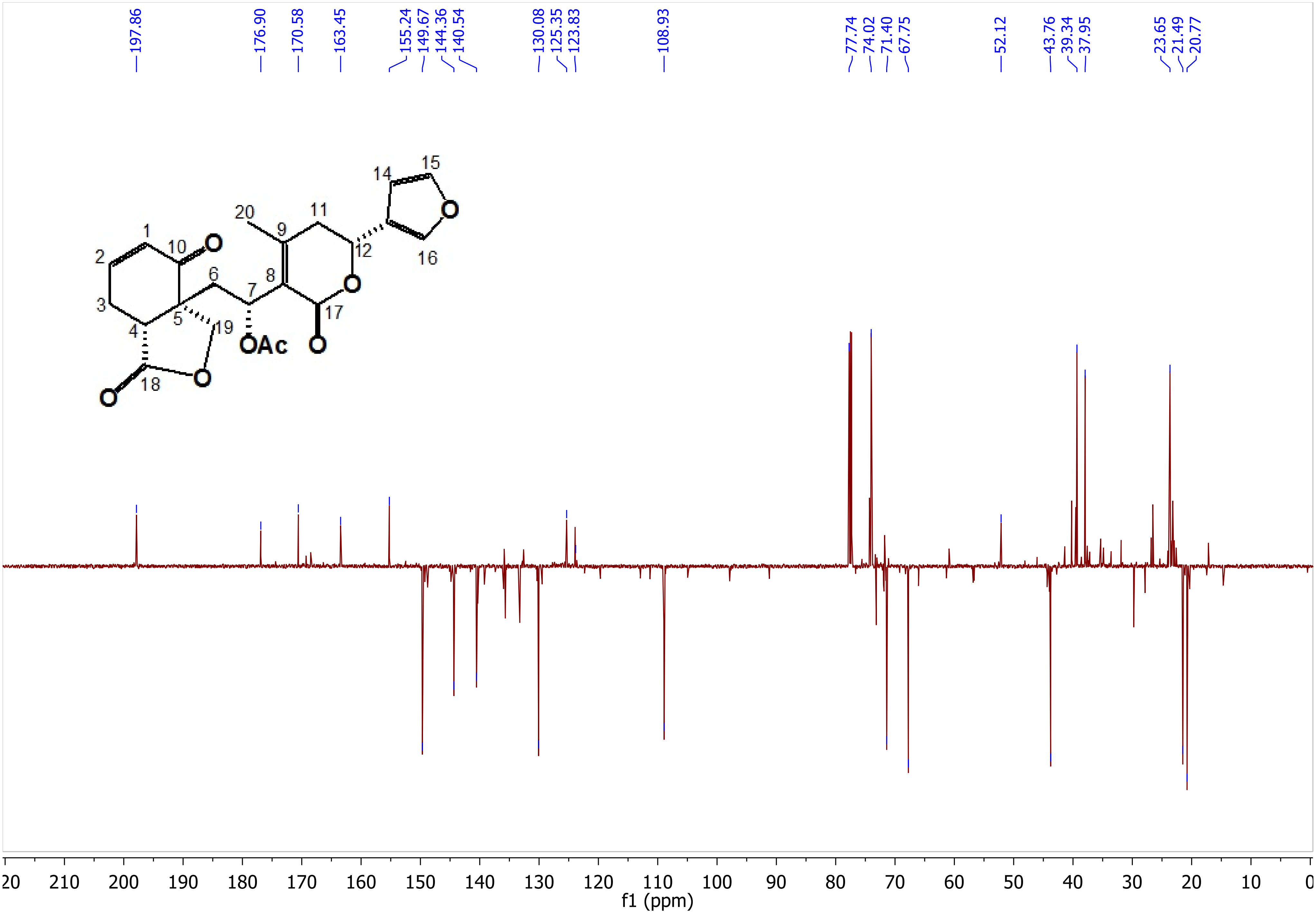

Figure S9. ^{13}C (DEPT) NMR ($CDCl_3$, 150 MHz) of salviandulin A (**2**)

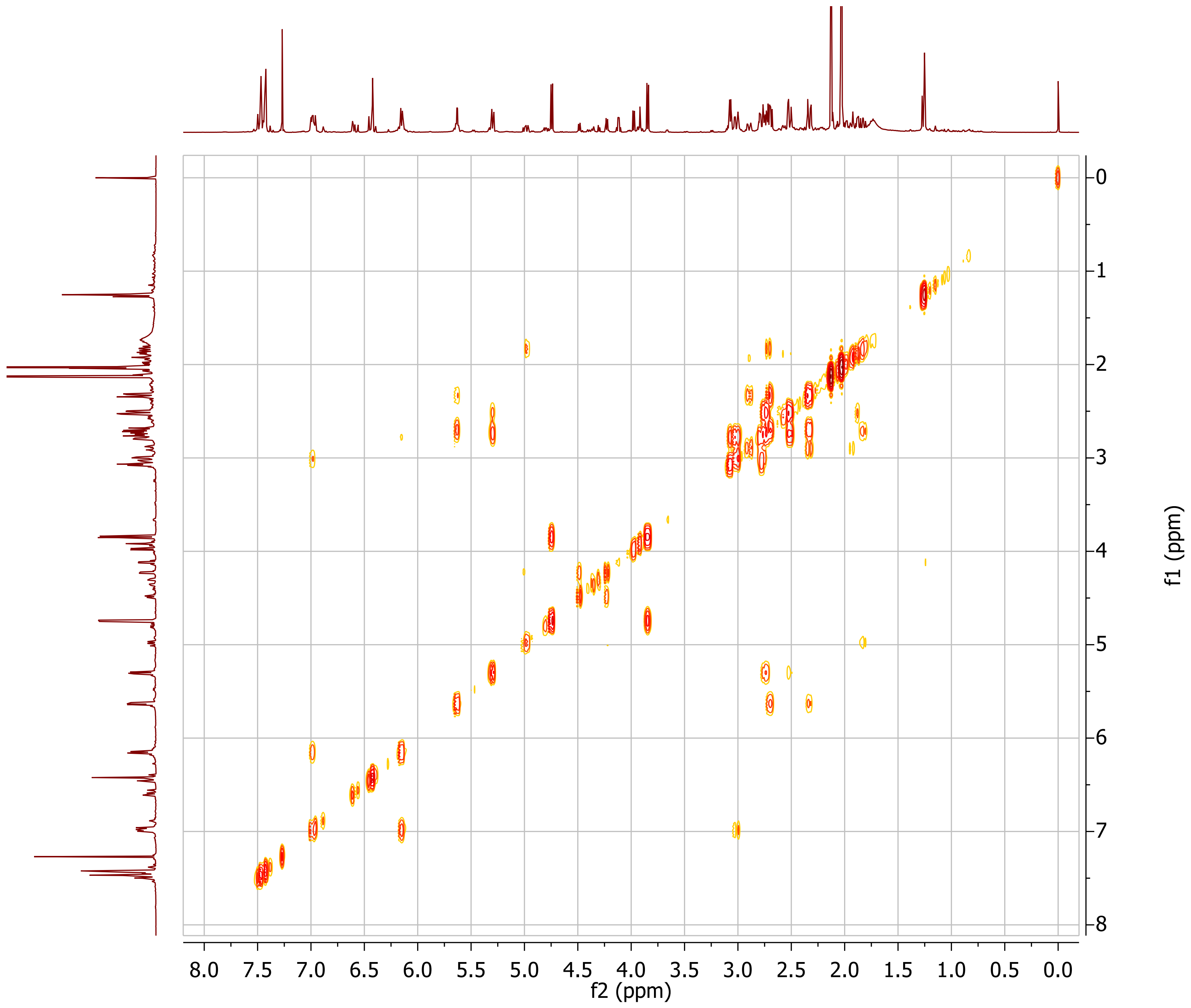

Figure S10. ^{1}H-^{1}H COSY NMR ($CDCl_3$, 600 MHz) of salviandulin A (**2**)

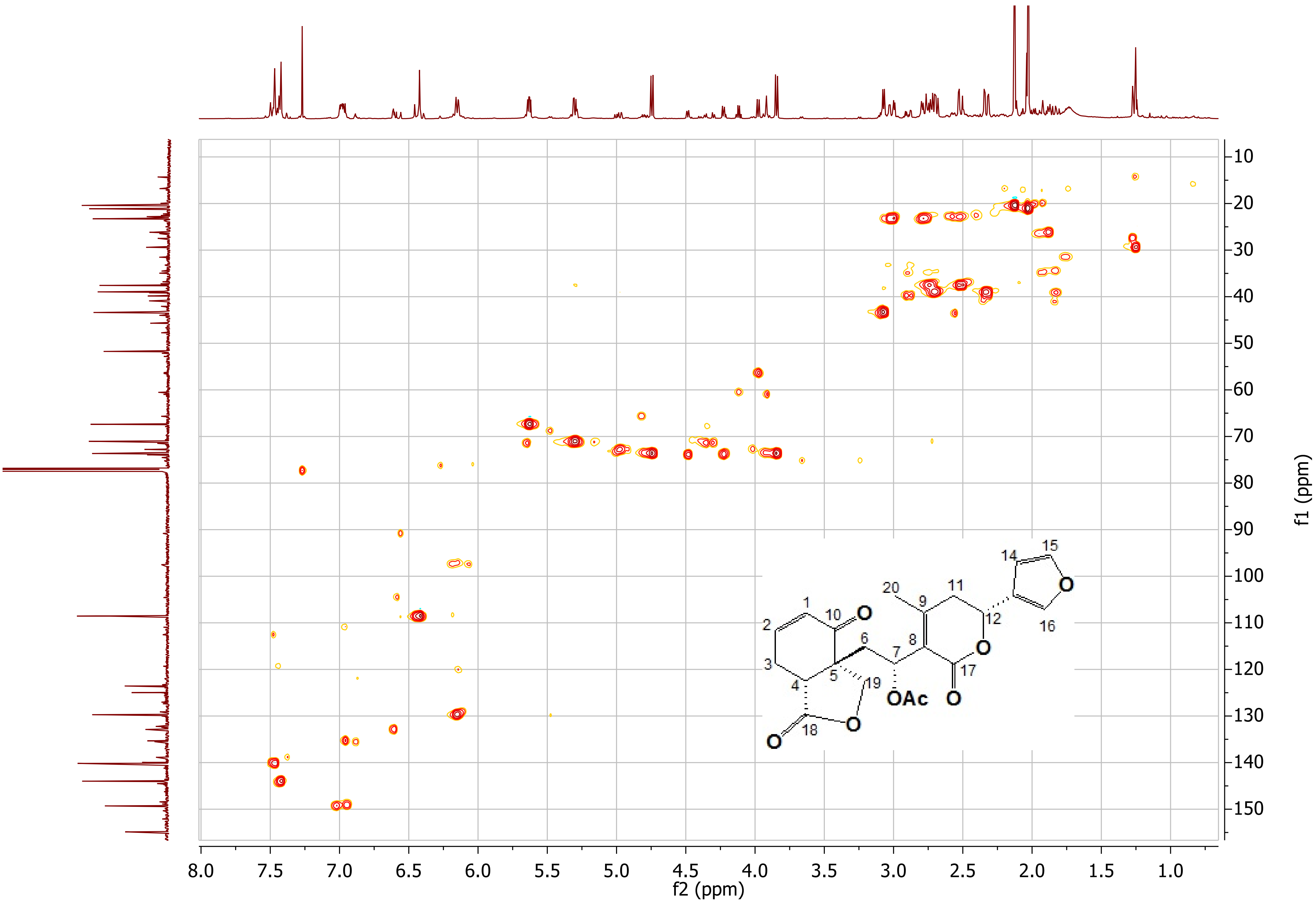

Figure S11. ^{1}H-^{13}C (HSQC) NMR ($CDCl_3$) of salviandulin A (**2**)

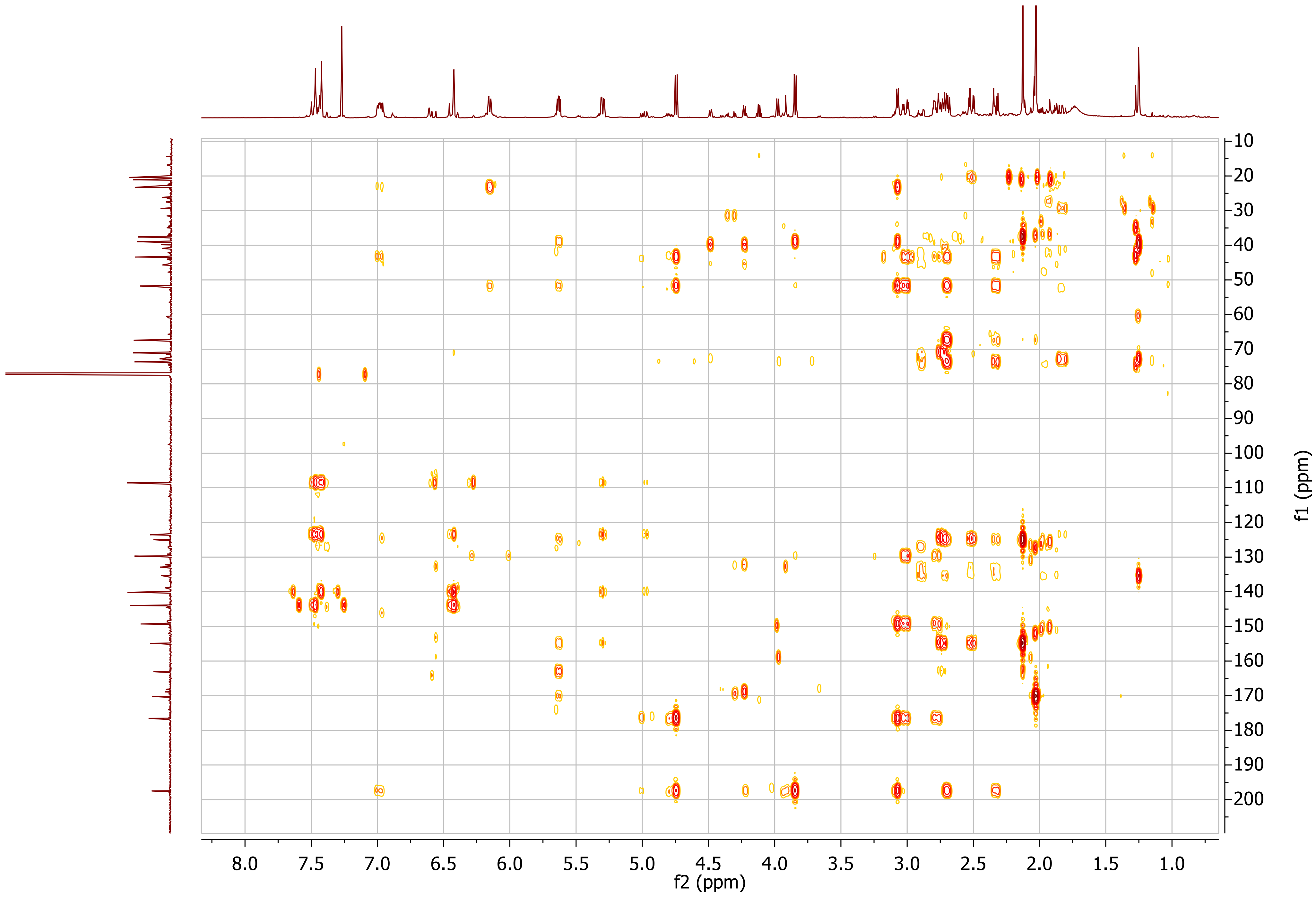

Figure S12. ^{1}H-^{13}C (HMBC) NMR ($CDCl_3$) of salviandulin A (**2**)

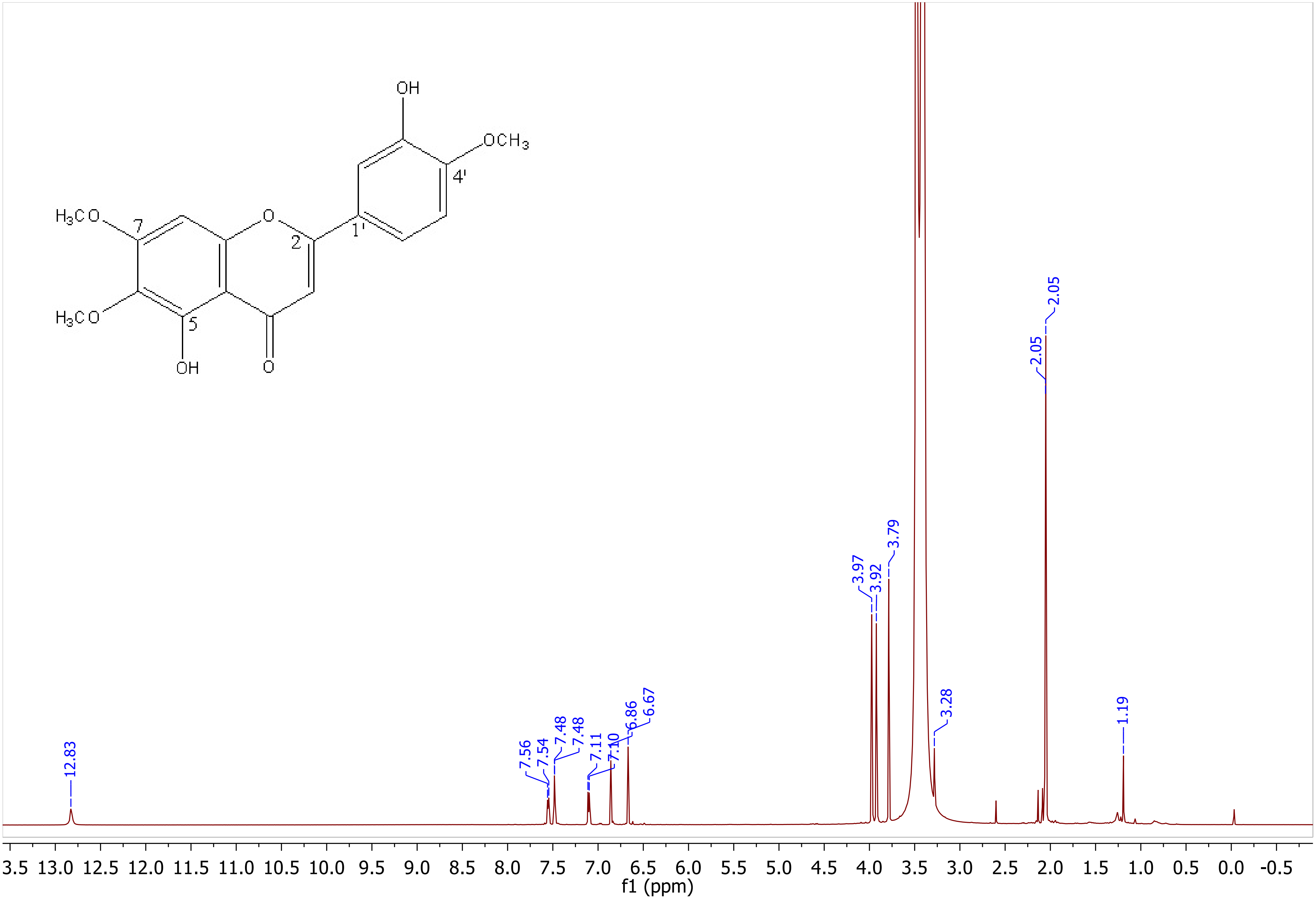

Figure S13. ^{1}H NMR (CD_3COCD_3, 600 MHz) of eupatorin (**3**)

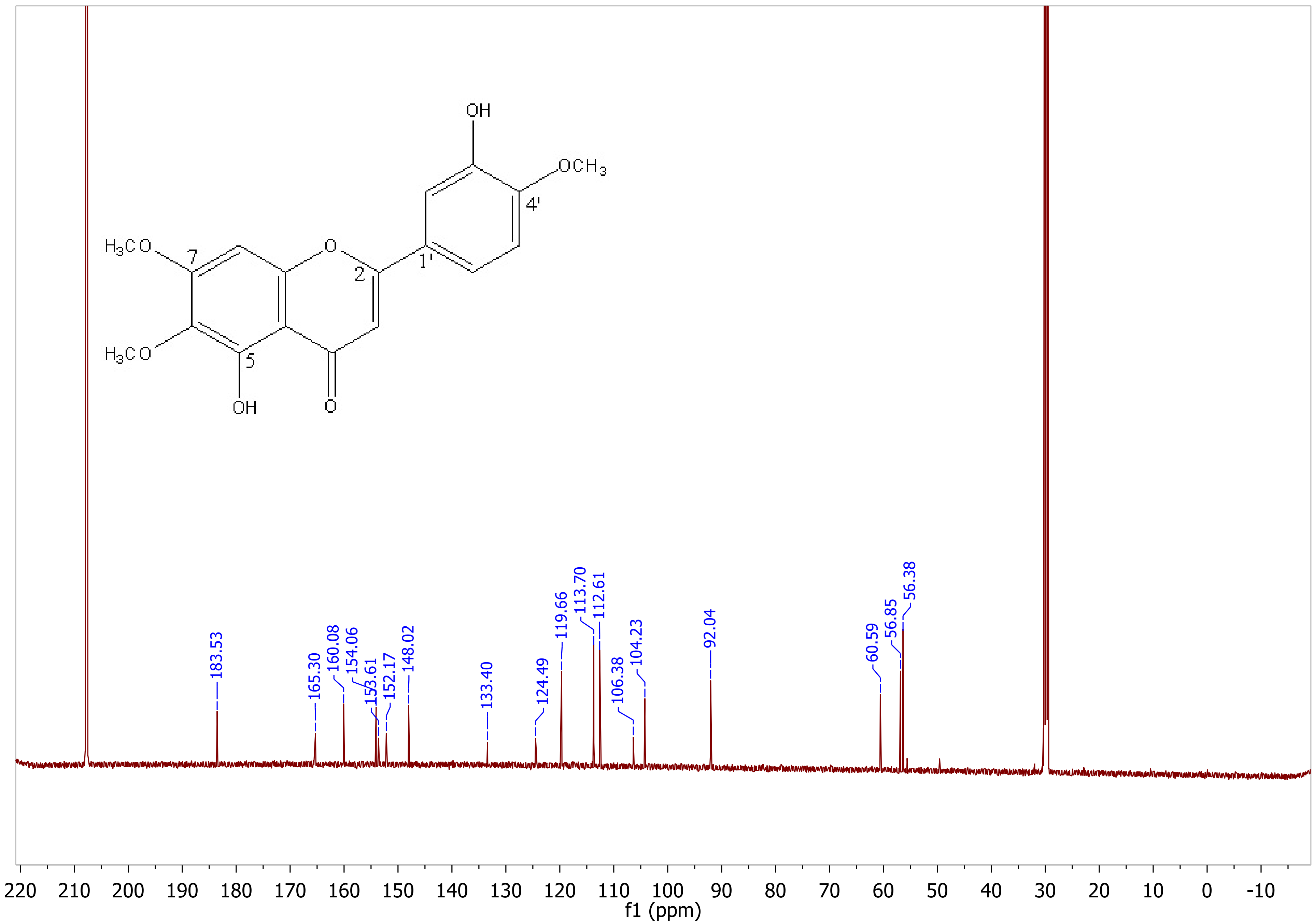

Figure S14. ^{13}C NMR (CD_3COCD_3, 150 MHz) of eupatorin (**3**)

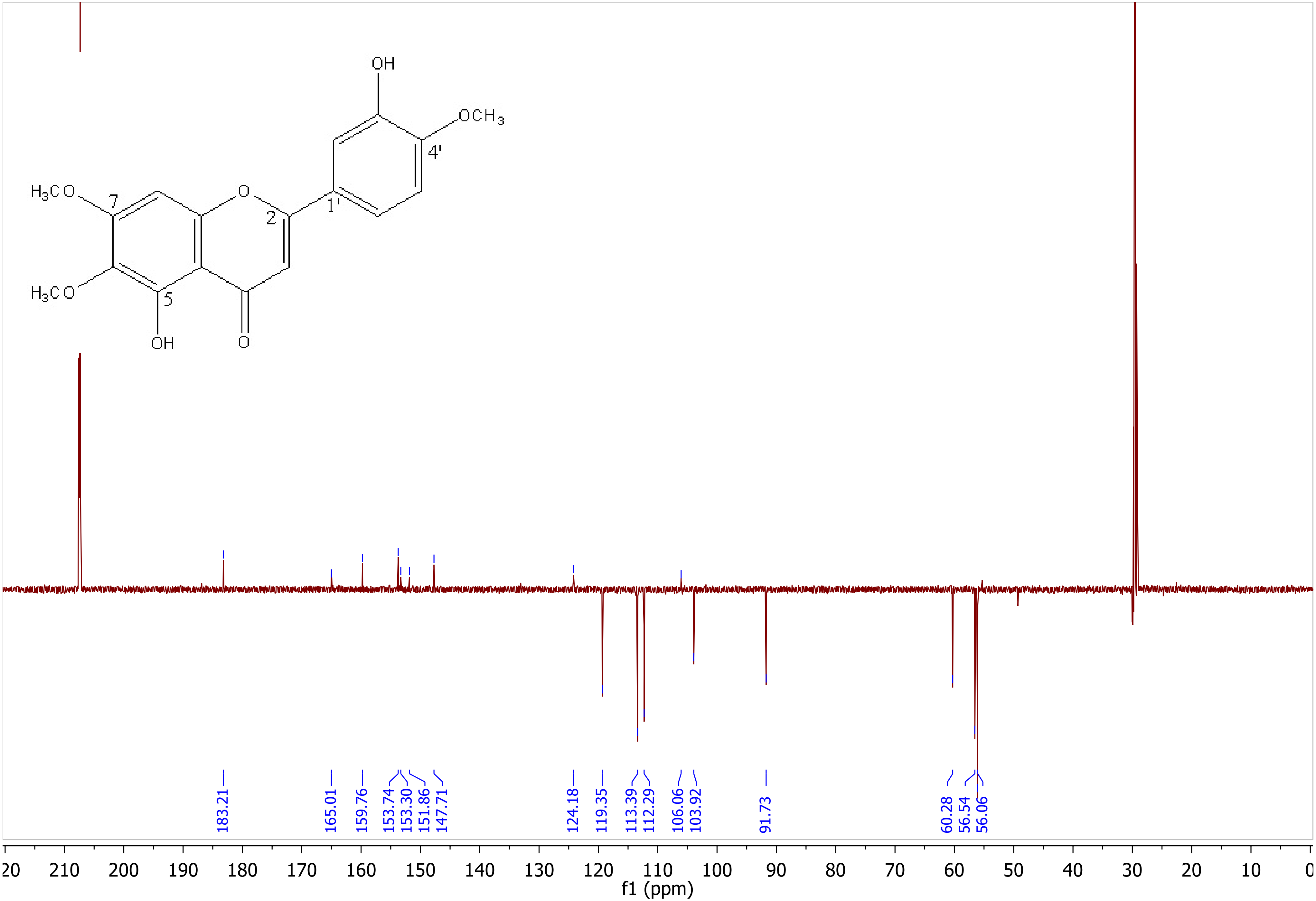

Figure S15. ^{13}C(DEPT) NMR (CD_3COCD_3, 150 MHz) of eupatorin (**3**)

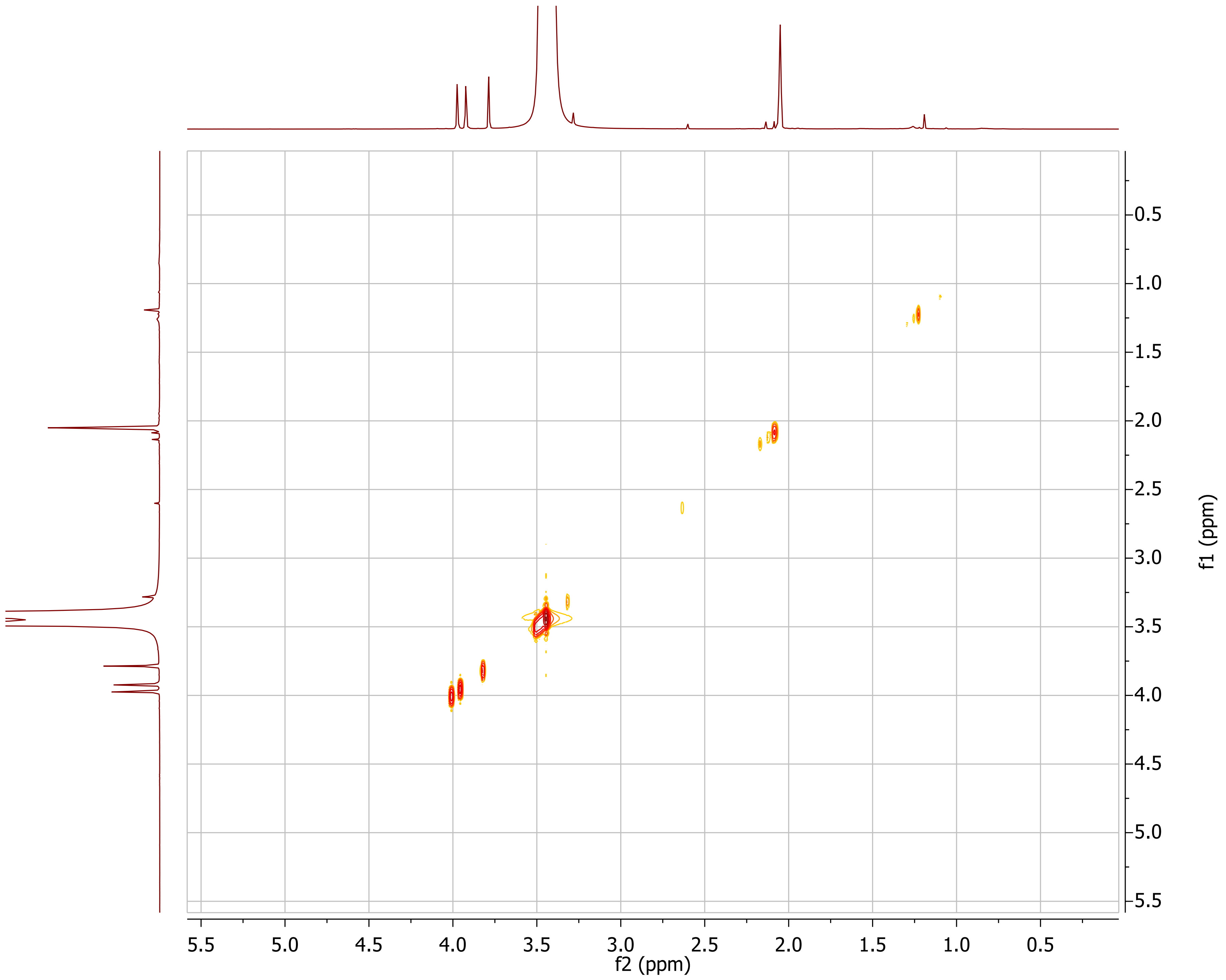

Figure S16. ^{1}H-^{1}H COSY NMR (CD_3COCD_3, 600 MHz) de eupatorin (**3**)

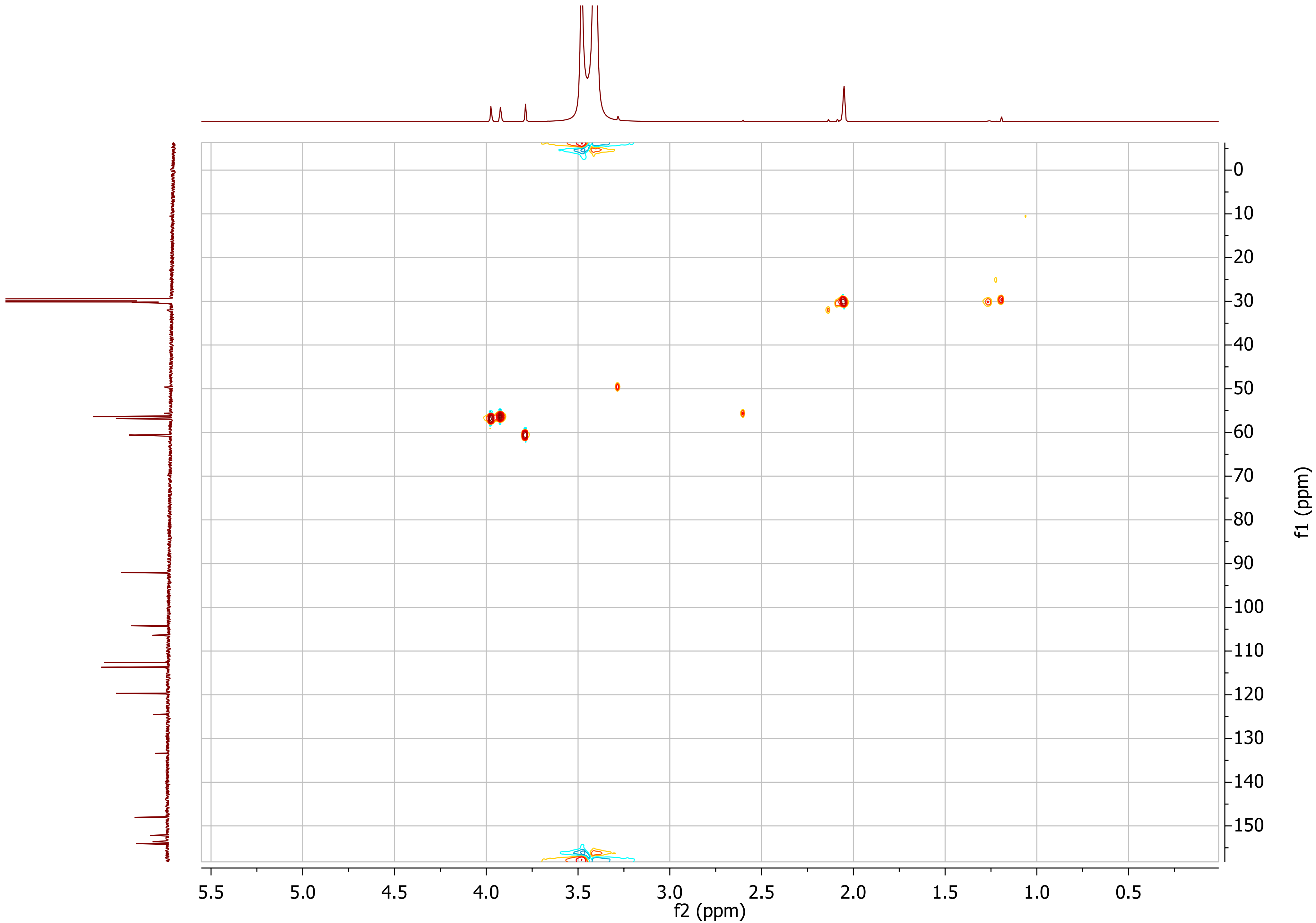

Figure S17. ^{1}H-^{13}C (HSQC) NMR (CD_3COCD_3, 600 MHz) de eupatorin (**3**)

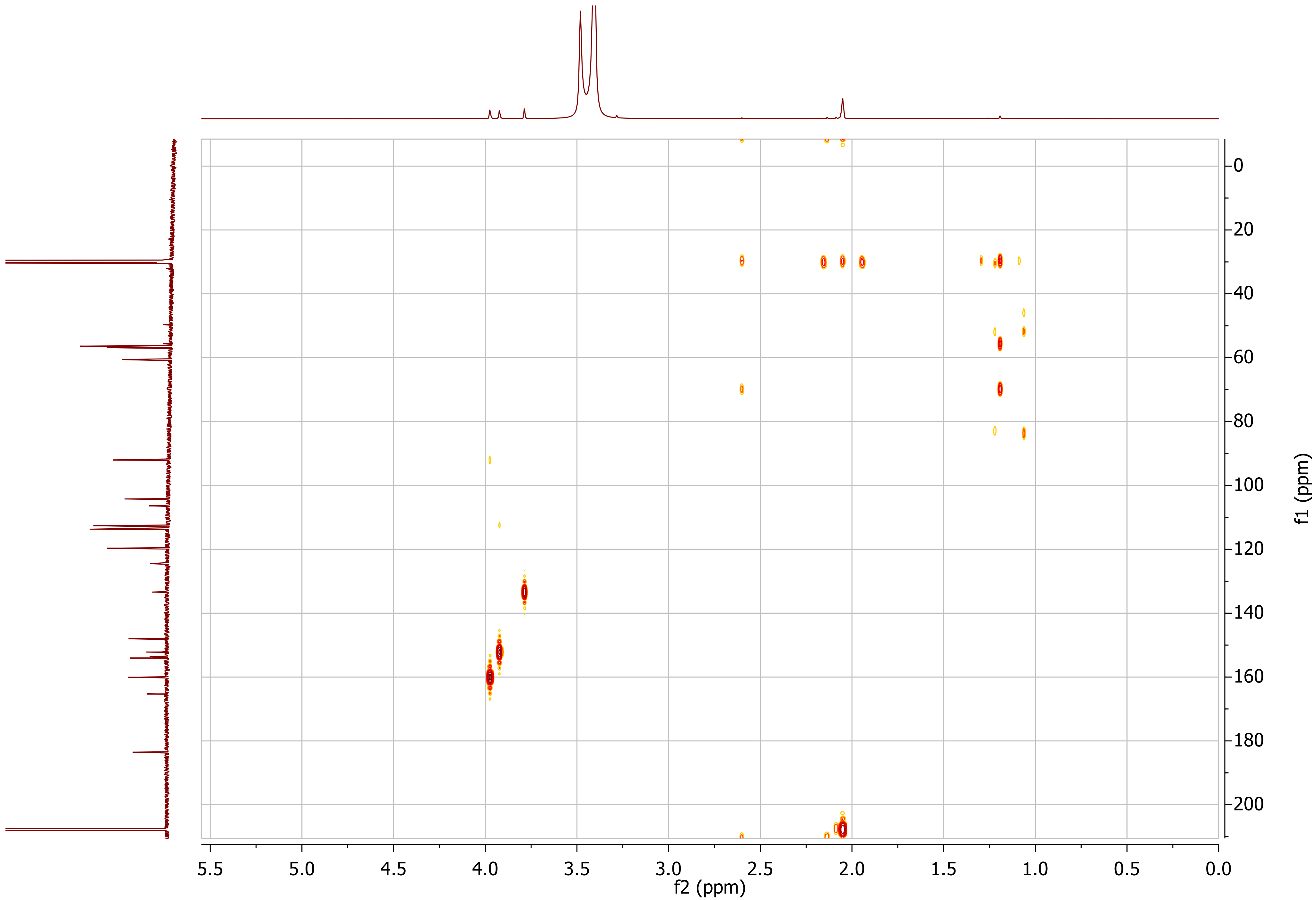

Figure S18. ^{1}H-^{13}C (HMBC) NMR (CD_3COCD_3, 600 MHz) de eupatorin (**3**)
